# Identification and characterization of the hydratase ThnL preparing for the second-ring cleavage in anaerobic naphthalene degradation

**DOI:** 10.1128/aem.00467-26

**Published:** 2026-09-23

**Authors:** Yachao Kong, Qiaohui Wang, Nadia A. Samak, Philip Weyrauch, Rainer U. Meckenstock

**Affiliations:** 1 Institute for Environmental Microbiology and Biotechnology, Aquatic Microbiology, University of Duisburg-Essen, Essen, Germany; 2 Key Laboratory of Coastal Biology and Bioresource Utilization, Yantai Institute of Coastal Zone Research, Chinese Academy of Sciences, Yantai, China; 3 College of Resource and Environment, University of Chinese Academy of Sciences 74519, Beijing, China; 4 State Key Laboratory of Bioreactor Engineering and School of Chemistry and Molecular Engineering, East China University of Science and Technology 47860, Shanghai, China; Universidad de los Andes, Bogotá, Colombia

**Keywords:** enoyl-CoA hydratase, naphthalene, sulfate-reducing bacteria, anaerobic degradation, polycyclic aromatic hydrocarbons

## Abstract

Anaerobic degradation of polycyclic aromatic hydrocarbons (PAHs) proceeds through CoA-activated intermediates and β-oxidation-like reactions; however, the enzymes responsible for key hydration and hydrolytic steps remain insufficiently defined. In the sulfate-reducing enrichment culture N47, the *thn* operon encodes multiple putative hydratases and hydrolases proposed to participate in anaerobic naphthalene degradation. Here, we cloned, heterologously expressed, and purified six candidate enzymes (ThnA, ThnH, ThnI, ThnL, ThnM, and ThnU) to elucidate their substrate specificities. Among them, ThnL showed robust activity toward 2-carboxycyclohexylideneacetyl-CoA, catalyzing rapid hydration of the enoyl-CoA double bond to form the tertiary alcohol 1-(1-hydroxy-2-carboxycyclohexyl)acetyl-CoA. ThnL exhibited kinetic parameters of Kₘ = 0.11 mM and Vₘₐₓ = 350 µmol min⁻¹ mg⁻¹. Two additional hydratases, ThnU and ThnI, converted the substrate only weakly (0.26% and 0.08% of ThnL activity), indicating promiscuous rather than physiological roles. ThnL also catalyzed the hydrolytic C1–C2 ring cleavage of 2-oxocyclohexane-1-carbonyl-CoA to pimelyl-CoA, revealing a bifunctional hydratase/hydrolase activity, although this reaction is unlikely to be physiologically relevant based on growth assays. This study shows that the ThnL-catalyzed hydration of 2-carboxycyclohexylideneacetyl-CoA to the tertiary alcohol 1-(1-hydroxy-2-carboxycyclohexyl)acetyl-CoA completes the final preparatory step before the second-ring cleavage in anaerobic naphthalene degradation.

**IMPORTANCE:**

Polycyclic aromatic hydrocarbons (PAHs), such as naphthalene, are widespread environmental pollutants that persist in oxygen-limited environments, such as marine sediments and contaminated aquifers. Although microorganisms are known to degrade these compounds anaerobically, the enzymatic mechanisms underlying key steps of these pathways remain poorly understood. In particular, the enzymes responsible for hydration reactions preceding ring cleavage in anaerobic naphthalene degradation have not been experimentally verified. In this study, we characterized several candidate enzymes encoded by the *thn* operon from a sulfate-reducing enrichment culture and identified ThnL as the enzyme catalyzing the hydration of 2-carboxycyclohexylideneacetyl-CoA. This finding clarifies a crucial preparatory step before the second-ring cleavage during anaerobic naphthalene metabolism and advances our understanding of microbial PAH degradation in anoxic environments.

## INTRODUCTION

Polycyclic aromatic hydrocarbons (PAHs), such as naphthalene, are ubiquitous environmental pollutants originating from natural and anthropogenic sources (1–3). Due to their hydrophobicity and recalcitrance, PAHs present a significant challenge for environmental remediation (4). While aerobic degradation of naphthalene has been extensively studied (5–7), the knowledge on anaerobic degradation pathways remains scarce (8).

In the absence of molecular oxygen, both mono- and poly-cyclic aromatic hydrocarbons are typically activated and converted into CoA-thioesters (9–11), which are reductively dearomatized and subsequently metabolized through a sequence of beta-oxidation-like reactions (12–14). This includes hydration of the remaining double bond(s), dehydrogenation, and hydrolytic ring cleavage, ultimately generating aliphatic acyl-CoA derivatives (15, 16). As such, hydratases and hydrolases are essential enzymes in the anaerobic degradation of aromatic compounds. Genomic and metabolite-based studies suggest that the ring-cleavage pathway in the degradation of naphthalene and phenanthrene also proceeds through beta-oxidation-like reactions (17, 18). However, the specific genes encoding hydratase and hydrolase enzymes involved in anaerobic naphthalene metabolism have not been fully identified yet.

In a previous study, the *thn* gene cluster in the genome of the sulfate-reducing enrichment culture N47 was verified to encode enzymes involved in the anaerobic degradation of naphthalene (19). The *thn* operon encodes seven putative hydratases/hydrolases, classified as crotonase-like hydratases (ThnA, ThnL, ThnM, and ThnU), MaoC-like dehydratases (ThnH and ThnI), and metallo-dependent hydrolases (ThnN) that might be involved in the lower pathway of anaerobic naphthalene degradation (20). In this study, we cloned and expressed the candidate genes encoding hydratase/hydrolase enzymes in *Escherichia coli*, purified the corresponding proteins, and elucidated the respective enzyme reactions.

## MATERIALS AND METHODS

### Growth of bacterial cells and preparation of cell extracts

The enrichment culture N47 (21) was cultivated anaerobically with artificial freshwater medium, naphthalene as the sole source of carbon and electrons, and sulfate as the electron acceptor, as described previously (17). A 1.6-L culture was incubated at 30°C for 6 weeks before harvesting by centrifugation at 3,200 × *g* and 4°C for 30 min (Centrifuge 5810 R, Eppendorf, Hamburg, Germany) in 500-mL airtight centrifuge beakers (Corning, Oneonta, USA). Cells were suspended in 100 mM MOPS (4-morpholinepropanesulfonic acid)/KOH buffer, pH 7.3, containing 15 mM MgCl_2_, operating inside a glove box (LabStar ECO, Garching, Germany) with an N_2_ atmosphere. Cell disruption was carried out anaerobically with a French press (Thermo Fisher, Dreieich, Germany) at 7 × 10^6^ Pa, as described previously (17). The cell extract was transferred to a 2-mL airtight reaction tube (Sarstedt, Nümbrecht, Germany) in the glove box and centrifuged at 18,200 × *g* for 90 min at 4°C to remove cell debris. The resulting supernatant was collected and used as the cell extract.

### Plasmid construction

Primers (Table S1) for the amplification of the genes *thnA* (N47_E41500), *thnH* (N47_E41420), *thnI* (N47_E41410), *thnL* (N47_E41380), *thnM* (N47_E41370), *thnN* (N47_E41360), and *thnU* (N47_E41300), which were designated as ORF28, ORF35, ORF36, ORF39, ORF40, ORF41, and ORF47 previously (22), were derived from GenBank sequence no. FR695877.1 (23). The genes *thnA*, *thnH*, *thnI*, *thnL*, *thnN*, and *thnU* were linked to a 6x-His tag at the C-terminus, and *thnM* with a Twin-Strep-tag at the C-terminus. Phusion Hot Start II High-Fidelity DNA Polymerase (Thermo Fisher Scientific, Ulm, Germany) was employed to amplify the target genes. PCR was performed using the following program: initial denaturation at 98°C for 30 s; then 30 cycles of 98°C for 10 s, annealing at the primer-specific temperature (Table S1) for 30 s, followed by 72°C for 1 min; and a final extension at 72°C for 5 min. The amplicons were ligated with the vector pETM-13 (European Molecular Biology Laboratory, EMBL, Heidelberg, Germany) and pASG-IBA103 (IBA Lifesciences, Göttingen, Germany) following the manufacturer’s protocol.

### Gene expression and purification

The His-tag fusion vectors containing target genes were transformed into *E. coli* Rosetta 2(DE3) (Merck Millipore, Darmstadt, Germany). After overnight growth at 37°C and 200 rpm in LB medium containing 100 µg mL^−1^ kanamycin and 34 µg mL^−1^ chloramphenicol, induction was carried out by transferring pre-cultures into auto-induction medium ZYM-5052 (24) and adjusting the incubation parameters to 20°C and 120 rpm at OD_600nm_ of 0.4–0.6. Cultures were incubated overnight, and bacteria were subsequently harvested by centrifugation for 30 min at 3,200 × *g* and 4°C using a 5810 R centrifuge (Eppendorf, Hamburg). The bacterial pellet was resuspended in 8 mL of 50 mM sodium phosphate buffer at pH 8.0 containing 300 mM NaCl and 5 μM imidazole and disrupted with a French Press (8 × 10^6^ Pa, Thermo Fisher, Dreieich, Germany). The cell lysate was centrifuged at 18,200 × *g* for 30 min (4°C) before applying the supernatant to a His GraviTrap TALON column (Cytiva, Amersham, UK) according to the manufacturer’s instructions. Unbound protein was removed by washing the column with 10 mL of 20 mM imidazole. His-tag fusion proteins were eluted with 3 mL of elution buffer containing 150 mM imidazole. The imidazole was removed afterward with PD-10 desalting columns (Cytiva).

*E. coli* BL21(DE3) (New England Biolabs, Frankfurt am Main, Germany) was used for expression of the Twin-Strep-tag fusion protein ThnM. Expression of the gene *thnM* was performed in LB medium (25) at 22°C and 130 rpm overnight; induction was carried out by adding 0.2 μg mL^−1^ anhydrotetracycline at OD_600nm_ of 0.4–0.6. The overexpressed ThnM protein was purified at 4°C by Strep-Tactin column (IBA Lifesciences) according to the manufacturer’s instructions. The ThnM protein was eluted with elution buffer containing 100 mM Tris/HCl, pH 8.0, 150 mM NaCl, 1 mM EDTA, and 50 mM biotin.

### Determination of the molecular mass of proteins

The purified proteins were separated by 12% sodium dodecyl sulfate-polyacrylamide gel electrophoresis (SDS-PAGE, Bio-Rad, Feldkirchen, Germany) to analyze the purity. To determine the molecular mass of the native protein complex of ThnL, 500 μL of purified His-tagged protein ThnL at a concentration of 1.8 mg/mL was loaded onto a Superose 6 gel filtration column (10 × 300 mm, Cytiva) connected to an ÄKTA purifier (GE Healthcare, Uppsala, Sweden). Elution was performed with 50 mM Tris/HCl buffer, pH 8.0, and 250 mM NaCl at a flow rate of 0.5 mL min^−1^, with absorbance monitored at 280 nm. The molecular mass calibration curve was obtained from the retention times of the reference size standards (catalog numbers 28403841 and 28403842; Cytiva).

### Synthesis and purification of CoA-thioesters

Racemic *cis*-isomer (1*R*,2*R*/1*S*,2*S*) of 2-(carboxymethyl)cyclohexane-1-carboxylic acid was synthesized as described previously (17). The synthesis of 5,6,7,8-tetrahydro-2-naphthoyl-CoA (THN-CoA), the CoA thioesters of racemic *cis-2-*(carboxymethyl)cyclohexane-1-carboxylic acid, 2-oxocyclohexane-1-carbonyl-CoA, and pimelyl-CoA was achieved through carbonyldiimidazole activation of the free acids, as detailed in reference 19. Target CoA-thioesters were purified using Chromabond C18 Hydra SPE columns (Macherey-Nagel, Düren, Germany); fractions were eluted with 2 mL 20% (vol/vol) acetonitrile in 0.1% (vol/vol) formic acid and analyzed with LC-MS as described below.

**Fig 1:**
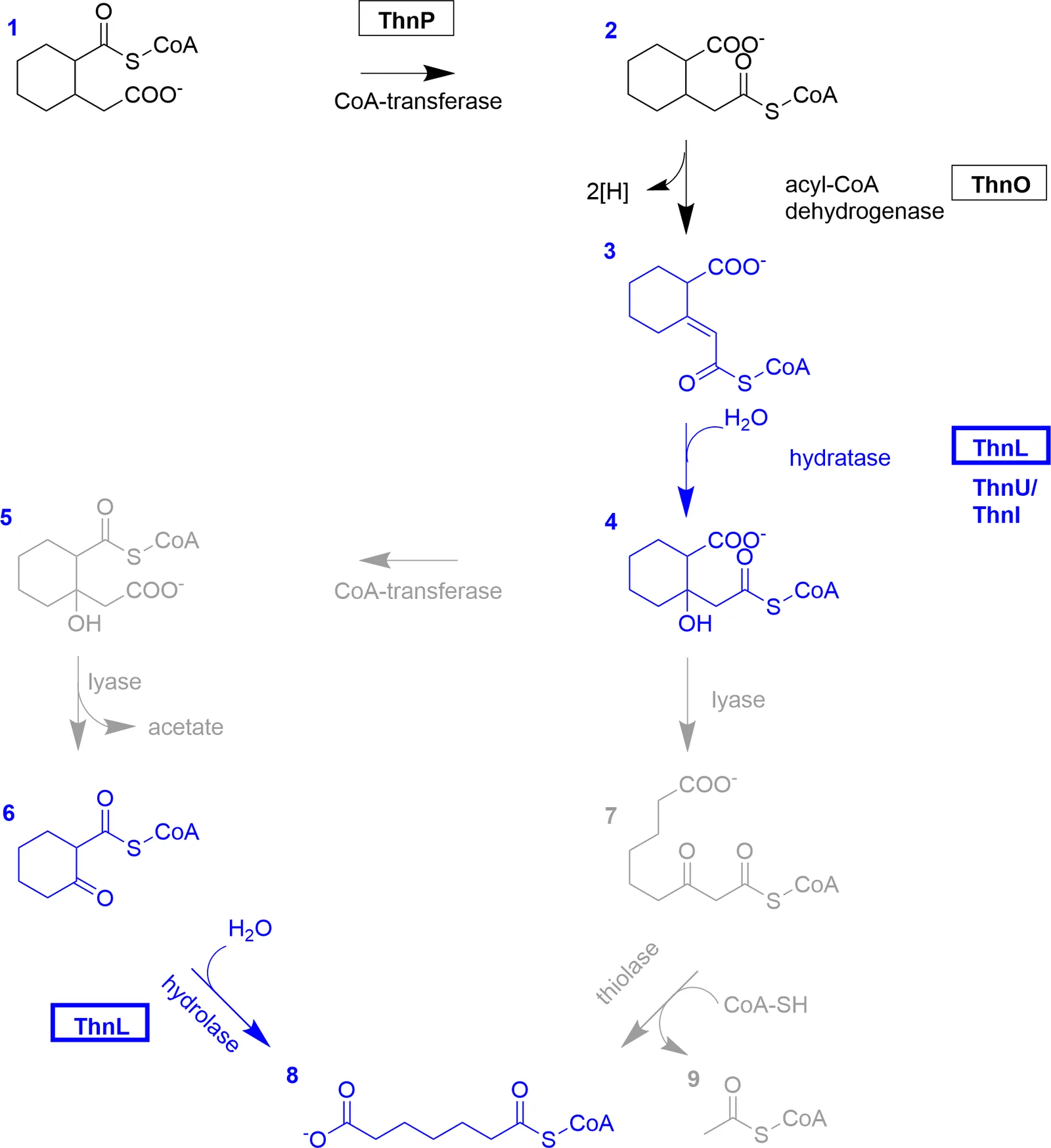
Proposed lower anaerobic naphthalene degradation pathway in strain N47, starting with 2-carboxymethyl-cyclohexane-carboxyl-CoA (1). Proven compounds, reactions, and enzymes described in previous studies (19) are shown in black, while compounds, reactions, and enzymes identified in this study are highlighted in blue. ThnL is shown in a separate blue box to distinguish it from isoenzymes ThnU and ThnI with much lower activity. Hypothetical reactions and compounds are shown in gray. Compounds are numbered as follows: (1) 2-carboxymethyl-cyclohexane-carboxyl-CoA, (2) 2-carboxycyclohexylacetyl-CoA, (3) 2-carboxycyclohexylideneacetyl-CoA, (4) 1-(1-hydroxy-2-carboxycyclohexyl)acetyl-CoA, (5) 2-hydroxy-2-(carboxymethyl)cyclohexane-1-carboxyl-CoA, (6) 2-oxocyclohexane-1-carbonyl-CoA, (7) 3-oxoazeloyl-CoA, (8) pimelyl-CoA, and (9) acetyl-CoA.

2-carboxycyclohexylideneacetyl-CoA (S-(2-(2-carboxycyclohexylidene)acetyl)-coenzyme A) was enzymatically synthesized by adding 0.4 mM of CoA-thioester of racemic *cis-2-*(carboxymethyl)cyclohexane-1-carboxylic acid to a combination of 36 μg of purified CoA-transferase ThnP and 45 μg of acyl-CoA dehydrogenase ThnO, which have been shown in a previous study to convert (1*R*,2*R*)−2-(carboxymethyl)cyclohexane-1-carboxyl-CoA to 2-carboxycyclohexylideneacetyl-CoA (19) (Fig. 1); 0.1 mM ferrocenium hexafluorophosphate and 0.1 mM phenazine methosulfate were used as artificial electron acceptors and redox mediators for the dehydrogenase reaction. A total of 600 μL reaction mixture was incubated in 1.5-mL reaction tubes (Sarstedt) for 30 min at 900 rpm and 30°C in a ThermoMixer C (Eppendorf, Hamburg, Germany) in the glove box; 30 μL of samples was taken immediately after the addition of enzymes as well as after 30 min of reaction, mixed with 60 μL of methanol, and centrifuged in an Eppendorf centrifuge for 45 min at 18,200 × *g* and 4°C to remove precipitated proteins and salts. The supernatant was then analyzed with LC-MS to assess enzymatic activity. The reaction was terminated by transferring the remaining mixture to a 2-mL reaction tube and adding a double volume (1,080 μL) of methanol. The proteins precipitated and were pelleted by centrifugation at 18,200 × *g* and 4°C for 45 min. The supernatant was concentrated in a rotational vacuum concentrator (RVC 2-25, Martin Christ, Osterode, Germany) to remove methanol and lyophilized in a freeze-dryer (Alpha 2-4 LSC, Martin Christ). The powder, containing the product 2-carboxycyclohexylideneacetyl-CoA, was stored directly at −20°C for further use without additional purification steps.

The intermediate hexahydro-2-naphthoyl-CoA (HHN-CoA) with m/z = 928 was synthesized anaerobically from tetrahydro-2-naphthoyl-CoA using cell extracts of strain N47 containing tetrahydro-2-naphthoyl-CoA reductase as described previously (14). HHN-CoA was formed in a reaction catalyzed by tetrahydro-2-naphthoyl-CoA reductase in the presence of NADH as electron donor. The 600 μL reaction contained 0.2 mM THN-CoA, 10 mM ATP, 5 mM NADH, and 50% (vol/vol) cell extracts (about 2.0–3.5 mg protein) in 100 mM MOPS/KOH buffer, pH 7.3, with 15 mM MgCl_2_. Samples were collected at 0 and 90 min for LC-MS analysis. Protein inactivation, concentration, and lyophilization of the metabolites were performed as outlined above. The lyophilized intermediates were stored at −20°C.

### Discontinuous *in vitro* assay

Enzyme activities were determined in discontinuous assays at 30°C and 900 rpm in a ThermoMixer C (Eppendorf) inside a glove box. The hydration of 2-carboxycyclohexylideneacetyl-CoA, hexahydro-2-naphthoyl-CoA, or the hydrolysis of 2-oxocyclohexane-1-carbonyl-CoA was followed by LC/MS analysis. Assays with heterologously produced enzymes were performed in 50 mM potassium phosphate buffer, pH 8.0. Instead, 100 mM MOPS/KOH buffer, pH 7.3, with 15 mM MgCl_2_ was used for assays using N47 cell extracts. The 200 μL reaction mixture typically contained one of the following: 60 μM 2-carboxycyclohexylideneacetyl-CoA, 0.2 mM 2-oxocyclohexane-1-carbonyl-CoA, or 0.4 mM hexahydro-2-naphthoyl-CoA. The heterologously produced enzymes ThnA, ThnH, ThnI, ThnL, ThnM, or ThnU were added to initiate the reactions. The added amounts of the respective proteins are detailed in the results section. To measure the activity of N47 cell extracts toward 2-carboxycyclohexylideneacetyl-CoA, reaction mixtures contained 800 μM 2-carboxycyclohexylideneacetyl-CoA and 10 μL N47 cell extracts (containing 41 μg protein).

The steady-state kinetics of hydratase ThnL was evaluated by measuring the conversion of 2-carboxycyclohexylideneacetyl-CoA over time. The reaction mixtures were prepared in a total volume of 300 μL with substrate concentrations ranging from 10 to 500 μM. Reactions were initiated by the addition of enzyme at a concentration of 0.5 μg/mL.

At various time points, a 30 μL sample was taken, to which a double volume of methanol was added to inactivate the enzyme and stop the reaction. Subsequently, the samples were centrifuged for 45 min at 18,200 × *g* and 4°C, after which the supernatant was subjected to LC-MS analysis. To determine the steady-state kinetic parameters of ThnL, data were analyzed using Michaelis-Menten kinetics, and Vmax and Km values were calculated using GraphPad Prism software.

### LC-MS analysis

Chromatographic separation was achieved with a Shimadzu high-performance liquid chromatography LC-2040C system and a Nucleodur C18 Gravity-SB column (100 × 3 mm, 5 μm particle size, Macherey-Nagel). A mobile phase composed of solvent A (0.1% [wt/vol] ammonium formate buffer, pH 6.5) and solvent B (acetonitrile) was applied, and elution followed a linear increase in solvent B from 5% to 35% over 15 min. The flow rate was set to 0.4 mL min^−1^, and the column temperature was maintained at 35°C.

Mass spectrometric detection was performed with an LC/MS-2020 single quadrupole mass spectrometer (Shimadzu) with an ESI system in positive ion mode with a mass-to-charge ratio (m/z) range of 50–1,050. Mass-to-charge ratios (m/z) of target ions corresponding to the substrate and product were monitored in single ion mode. Nebulizing gas flow was set to 1.5 L min^−1^, and drying gas flow was set to 12 L min^−1^.

### Sequence alignment, phylogenetic analysis, and homology modeling

The amino acid sequences of the open reading frames of hydratases/hydrolases within the *thn*-operon were compared with those analogs present in the database using the BLAST algorithm at the UniProt server (https://www.uniprot.org). Pairwise and multiple sequence alignments were conducted with the MUSCLE program (26) using MEGA11 software (27). The evolutionary analysis was inferred using the Neighbor-Joining method (28) and was conducted in MEGA11 software. The AlphaFold structures of ThnA, ThnL, ThnM, and ThnU were retrieved from the UniProt database. The PyMOL software was used to visualize the structure models.

## RESULTS

### Production and size characterization of recombinant protein

**Fig 2:**
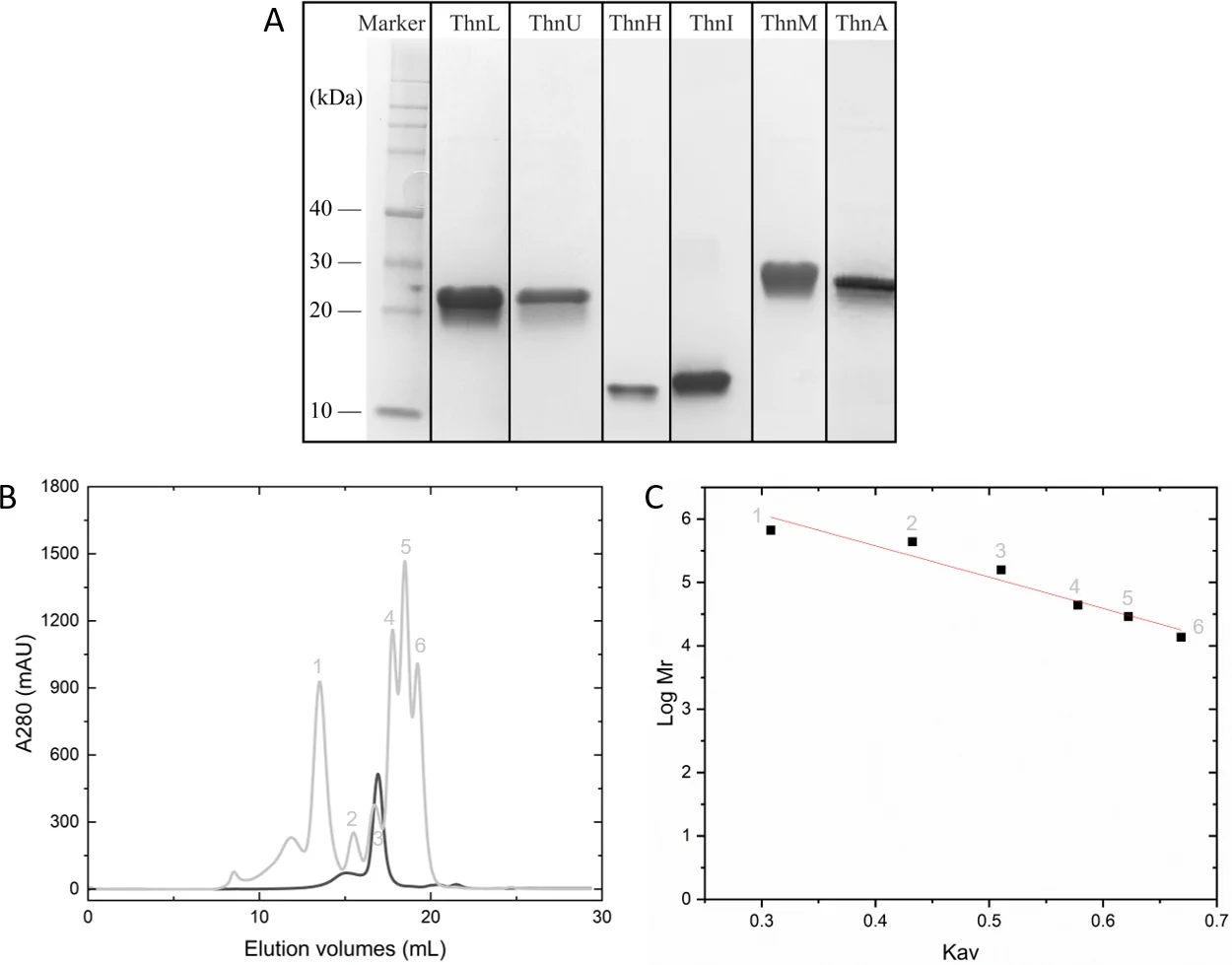
Heterologous production and purification of recombinant hydratases/hydrolases ThnA, ThnH, ThnI, ThnL, ThnM, and ThnU, and determination of complex size of ThnL. (**A**) 12% SDS-PAGE of purified hydratases/hydrolase ThnL/ThnU/ThnH/ThnI/ThnM/ThnA; 4.7 μg of ThnL, 2.8 μg of ThnU, 2.7 μg of ThnH, 8.0 μg of ThnI, 4.2 μg of ThnM, and 4.0 μg of ThnA were used for SDS-PAGE. Numbers on the *Y*-axis indicate the molecular masses of standard proteins in lane “Marker.” (**B**) Size exclusion chromatography of the purified ThnL complex (black line) overlaid with the molecular mass standards (gray line). The molecular mass of ThnL was determined to be 93 kDa after comparison with the calibration curve. (**C**) Calibration curve generated by plotting Kav against log Mr using the indicated molecular mass standards. The calibration proteins are labeled as 1, Thyroglobulin; 2, Ferritin; 3, Aldolase; 4, Ovalbumin; 5, Carbonic anhydrase; and 6, Ribonuclease A.

Seven genes putatively encoding hydratase or hydrolase enzymes within the *thn* operon were expressed in *E. coli*. Except for ThnN, which was produced in an insoluble form, all recombinant proteins tagged with His6 or Twin-strep tags were obtained as soluble enzymes and subsequently purified to homogeneity via affinity chromatography (Fig. 2). SDS-PAGE analysis of the purified proteins showed protein bands roughly consistent with the predicted monomeric molecular masses of ThnA, ThnH, ThnI, ThnL, ThnM, and ThnU, which were calculated to be 30.0, 17.7, 17.4, 29.3, 32.6, and 31.3 kDa, respectively (Fig. 2A). The oligomeric state of ThnL was further analyzed by size-exclusion chromatography, which yielded an apparent molecular mass of approximately 93 kDa for the major peak of the native protein, indicating that ThnL most likely adopts a homotrimeric assembly in solution (Fig. 2B). The apparent molecular mass was estimated from the calibration curve shown in Fig. 2C. This oligomeric state is consistent with previous reports showing that members of the crotonase superfamily commonly exist as trimers or hexamers (dimers of trimers) (29). A minor peak eluting at approximately 15 mL, corresponding to an apparent molecular mass of >400 kDa, was also observed and most likely represents a small population of impurities or aggregated ThnL species formed during sample storage. The native molecular masses of the other proteins were not investigated, as they did not specifically catalyze the target reactions.

### Substrate conversion with recombinant enzymes

To assign the potential hydratase and hydrolase genes within the *thn* operon to specific hydration or hydrolysis reactions in the anaerobic naphthalene degradation pathway, we investigated the ability of these candidate enzymes to degrade relevant metabolites.

#### Conversion of 2-carboxycyclohexylideneacetyl-CoA

2-carboxycyclohexylideneacetyl-CoA, a product of the dehydrogenation of 2-carboxycyclohexylacetyl-CoA, was enzymatically synthesized from the CoA thioesters of the racemic *cis*-isomer (1*R*,2*R*/1*S*,2*S*) of *2-*(carboxymethyl)cyclohexane-1-carboxylic acid with CoA-transferase ThnP and acyl-CoA dehydrogenase ThnO (Fig. 1) (19). Given the specificity of ThnO toward (1*R*,2*R*)-2-carboxycyclohexylacetyl-CoA, only 50% of the CoA thioesters of the racemic *cis-2-*(carboxymethyl)cyclohexane-1-carboxylic acid were converted to 2-carboxycyclohexylideneacetyl-CoA, which was subsequently used for the identification of potential hydratases.

**Fig 3:**
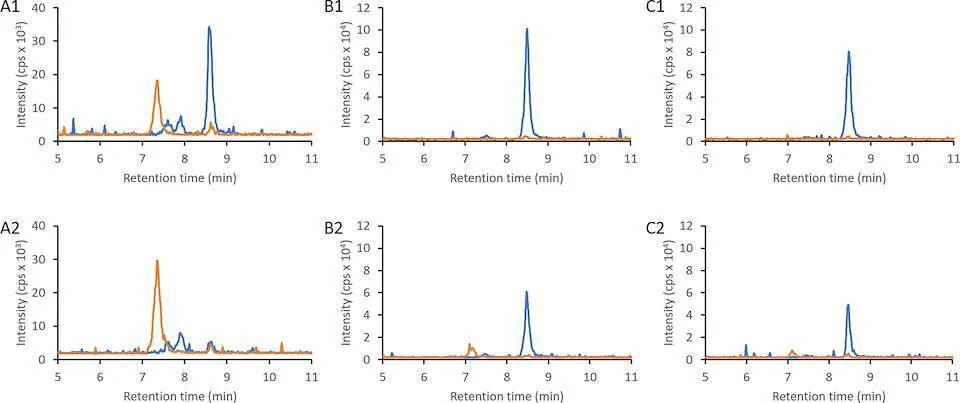
LC-MS chromatograms showing the conversion of 2-carboxycyclohexylideneacetyl-CoA (m/z = 934, blue lines) to 2-(1-hydroxy-2-carboxycyclohexyl)acetyl-CoA (m/z = 952, orange lines) by heterologously expressed hydratases ThnL (**A**), ThnU (**B**), and ThnI (**C**). (**A1**) Conversion of substrate with ThnL, *t* = 0 min. (**A2**) *t* = 3 min. (**B1**) Conversion of substrate with ThnU, *t* = 0 min. (**B2**) 5 min. (**C1**) Conversion of substrate with ThnI, *t* = 0 min. (**C2**) *t* = 5 min.

**Fig 4:**
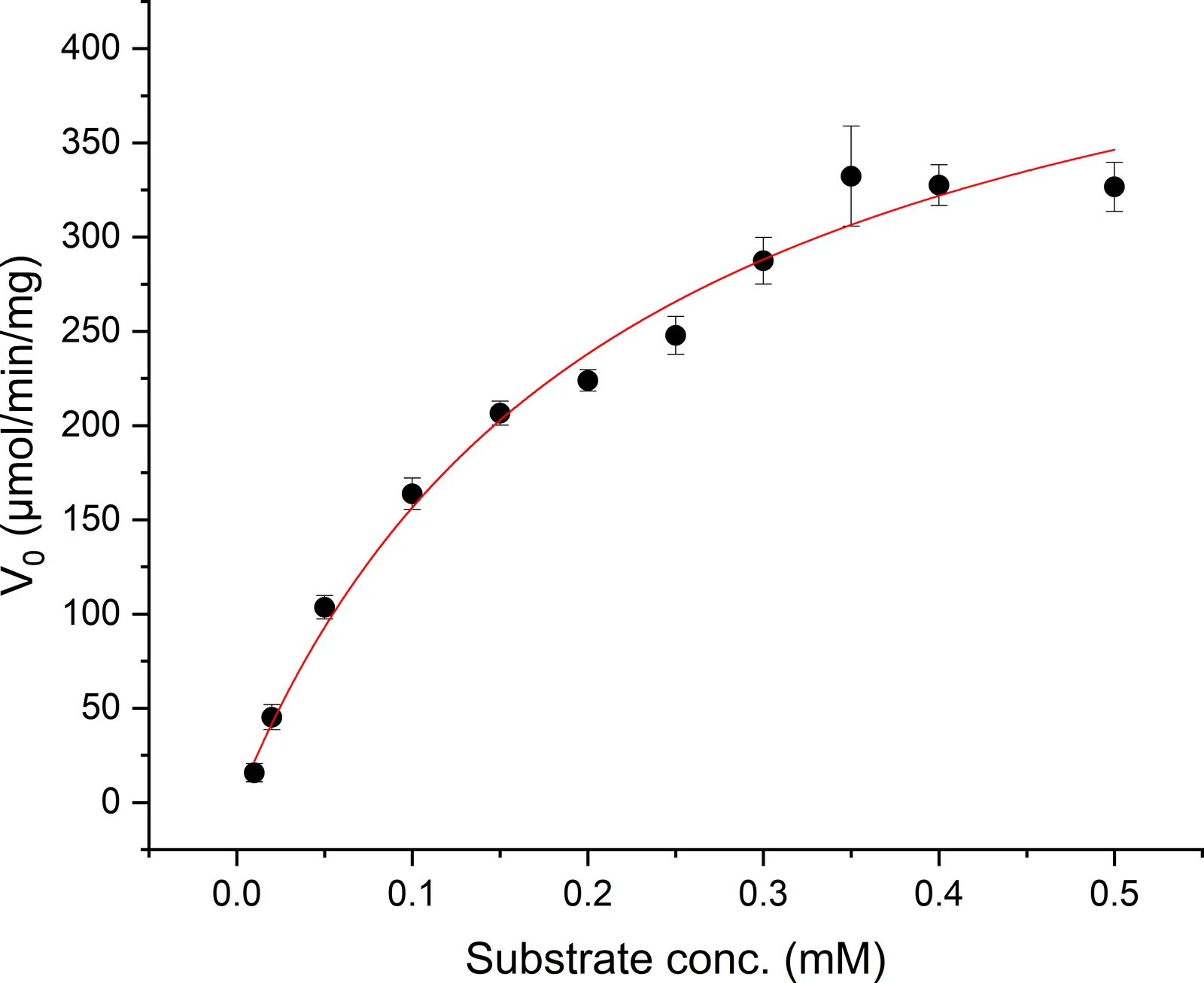
Michaelis-Menten plot of the reaction kinetics of ThnL incubated with various concentrations of the substrate 2-carboxycyclohexylideneacetyl-CoA.

Out of the six purified, recombinant potential hydratase or hydrolase enzymes (ThnA, ThnH, ThnI, ThnL, ThnM, and ThnU), only ThnL showed substantial catalytic activity toward 2-carboxycyclohexylideneacetyl-CoA, while only trace product formation was detected for ThnI and ThnU (Fig. 3). The observed mass difference of +18 units between the substrate (retention time 8.6 min in LC/MS, m/z 934) and the product (retention time 7.3 min in LC/MS, m/z 952) indicated the hydration of the double bond in the side chain of 2-carboxycyclohexylideneacetyl-CoA (Fig. 3), most likely producing the tertiary alcohol 1-(1-hydroxy-2-carboxycyclohexyl)acetyl-CoA (S-[(1-hydroxy-2-carboxycyclohexyl)acetyl]-coenzyme A). When 4 μg/mL of ThnL was added to 60 μM 2-carboxycyclohexylideneacetyl-CoA, 93% of the substrate (56 μM out of 60 μM) was converted within 3 min, corresponding to a specific activity of 4.7 μmol min^−1^ mg^−1^ protein. The kinetic parameters of ThnL for 2-carboxycyclohexylideneacetyl-CoA showed an apparent Km value of 0.11 mM, and a V_max_ of 350 μmol min^−1^ mg^−1^ (Fig. 4). Comparatively, reactions with the other two hydratases (ThnU and ThnI) that showed activity with 2-carboxycyclohexylideneacetyl-CoA containing either 392 μg/mL ThnU or 1766 μg/mL ThnI, respectively, resulted in only 40% or 57% of substrate conversion within 5 min, corresponding to specific activities of 0.012 and 0.004 μmol min^−1^ mg^−1^ protein, respectively. These findings indicate that ThnL is most likely the specific hydratase catalyzing the hydration of 2-carboxycyclohexylideneacetyl-CoA, whereas ThnU and ThnI act as promiscuous enoyl-CoA hydratases responsible for other reactions in the pathway. Despite its high specific activity, ThnL does not achieve complete substrate conversion, with a conversion rate of 96%; this may be due to chemical equilibrium, as enoyl-CoA hydratase catalyzes a reversible hydration/dehydration reaction.

#### Conversion of hexahydro-2-naphthoyl-CoA

Hexahydro-2-naphthoyl-CoA (m/z 928) was biologically synthesized using N47 cell extracts and used as a substrate in subsequent enzyme assays to identify candidate hydratases or hydrolases capable of converting it. However, none of the six tested enzymes showed detectable activity, as neither the expected product nor any additional product peaks were observed.

#### Conversion of 2-oxocyclohexane-1-carbonyl-CoA

**Fig 5:**
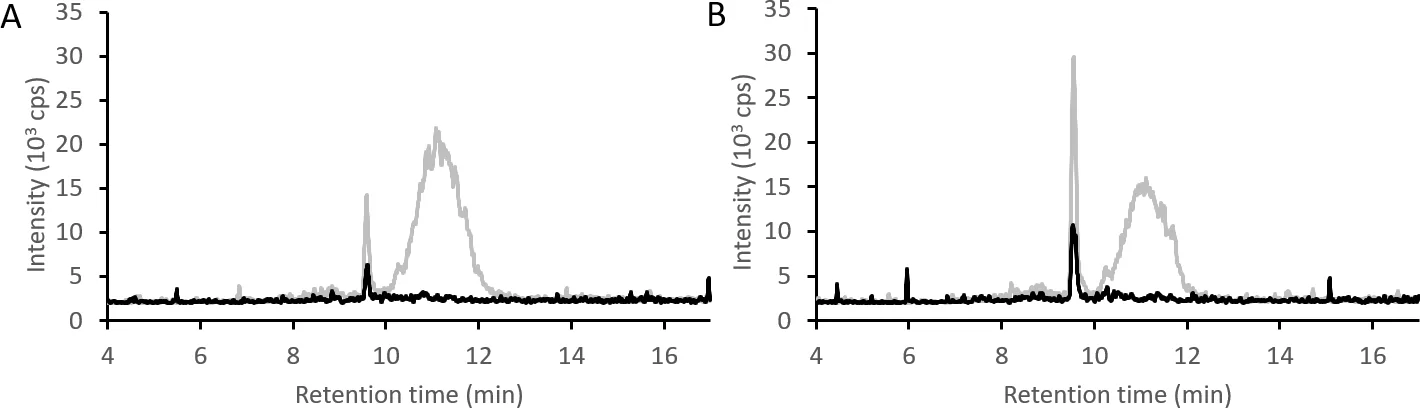
LC-MS chromatograms showing the conversion of 2-oxocyclohexane-1-carbonyl-CoA (m/z = 892, gray lines) to pimelyl-CoA (m/z = 910, black lines) by heterologously expressed ThnL. (**A**) Conversion of the substrate at *t* = 0 min. (**B**) *t* = 180 min.

The gene product ThnL was also the only enzyme among the six hydratase/hydrolase candidates that catalyzed the conversion of 2-oxocyclohexane-1-carbonyl-CoA (m/z = 892, retention time 11.1 min), achieving 36% substrate conversion within 180 min and a specific activity of 0.003 μmol min^−1^ mg^−1^ protein. The product peak in the LC/MS chromatogram was compared with a synthesized pimelyl-CoA standard with m/z = 910 (retention time 9.5 min), indicating that the reaction comprises a hydrolytic ring cleavage of the C1-C2 bond, producing pimelyl-CoA (Fig. 5). Hence, ThnL is not only a hydratase but is a bifunctional enzyme that has both hydratase and hydrolase activity. However, it is unclear if the hydrolase activity has a physiological function in anaerobic naphthalene degradation since the specific activity is very low.

### Substrate conversion by cell extracts

**Fig 6:**
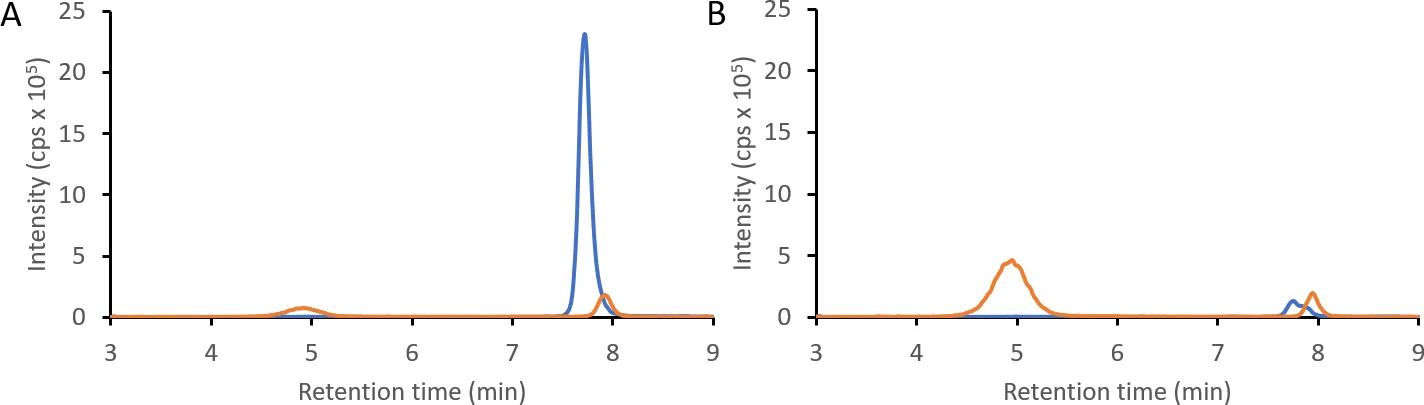
LC-MS chromatograms showing the conversion of 2-carboxycyclohexylideneacetyl-CoA (m/z = 934, blue line) to the tertiary alcohol 1-(1-hydroxy-2-carboxycyclohexyl)acetyl-CoA (m/z = 952, orange line) in cell-free extract of culture N47. (**A**) Conversion of the substrate 2-carboxycyclohexylideneacetyl-CoA at *t* = 0 min. (**B**) *t* = 2 min.

Cell extracts from naphthalene-grown N47 cells consumed 2-carboxycyclohexylideneacetyl-CoA at a rate of 0.056 μmol min^−1^ mg^−1^ protein, confirming that the reaction is indeed taking place in N47 cells (Fig. 6). The specific activity was quantified by following the formation of 1-(1-hydroxy-2-carboxycyclohexyl)acetyl-CoA, the immediate hydration product. A systematic shift in retention time was observed in this LC-MS run (Fig. 6), while compound identification was confirmed by the expected mass-to-charge ratios and the corresponding MS spectra. Under the assay conditions, only this hydration product accumulated, and no further metabolites were observed. Consequently, the measured specific activity represents the hydratase reaction in the extract solely, without contributions from downstream enzymes.

ThnL displayed measurable but weak activity toward 2-oxocyclohexane-1-carbonyl-CoA *in vitro*. However, when 2-oxocyclohexane-1-carboxylic acid was supplied as the sole source of carbon and electrons for strain N47, no significant microbial growth was observed after 2 months. This indicates that despite ThnL’s small ability to convert 2-oxocyclohexane-1-carbonyl-CoA under assay conditions, this transformation is unlikely to occur as a physiologically relevant step in the anaerobic naphthalene degradation pathway of N47.

## DISCUSSION

The anaerobic degradation of naphthalene is the first among the polycyclic aromatic hydrocarbons that has been elucidated to molecular detail. Through a series of *in vitro* assays with cell extracts of strain N47 cells grown on naphthalene and purified recombinant enzymes, we previously identified unknown enzymes involved in anaerobic naphthalene degradation (19, 30). In this study, we identified and characterized the bifunctional enoyl-CoA hydratase/acyl-CoA hydrolase ThnL encoded in the *thn* operon (23). The enzyme catalyzes the hydration of 2-carboxycyclohexylideneacetyl-CoA, producing the tertiary alcohol 1-(1-hydroxy-2-carboxycyclohexyl)acetyl-CoA. Additionally, two other enoyl-CoA hydratases encoded by genes *thnU* and *thnI* were found to catalyze the hydration of 2-carboxycyclohexylideneacetyl-CoA but with only 0.26% and 0.08% activity compared to ThnL. Therefore, ThnU and ThnI are hypothesized to be promiscuous enoyl-CoA hydratases likely involved in alternative reactions within the degradation pathway.

### Evolutionary analysis

**Fig 7:**
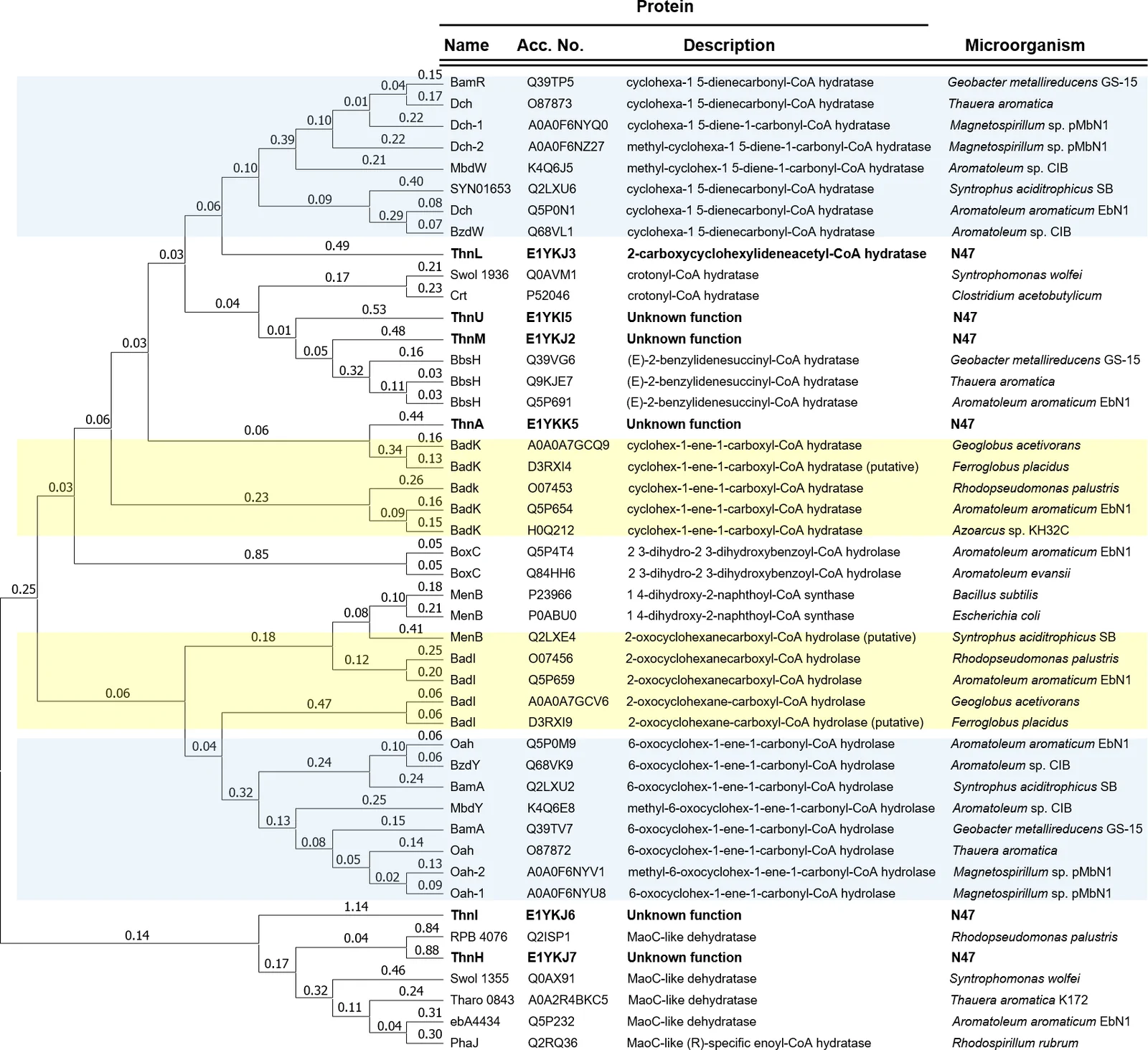
Phylogenetic tree (neighbor-joining) of enoyl-CoA hydratase/hydrolase ThnA, ThnH, ThnI, ThnL, ThnM, and ThnU encoded within the thn-operon and analogues. Protein names and UniProt codes are shown. Proteins encoded within the thn-operon are marked in bold. The Thauera-type and Rhodopseudomonas-type hydratases/hydrolases are indicated within blue and yellow boxes, respectively.

Phylogenetic analysis of the six hydratase/hydrolase candidates within the *thn* operon (Fig. 7) revealed that ThnA, ThnL, ThnM, and ThnU share high sequence identities (39%–47%) with crotonyl-CoA hydratases from the obligatory anaerobes *Clostridium acetobutylicum* and *Syntrophomonas wolfei* (31, 32). Crotonyl-CoA hydratase belongs to the crotonase superfamily, which is capable of catalyzing reactions ranging from alkene hydration/isomerization, (de)carboxylation, CoA thioester and peptide hydrolysis, and C-C bond formation, cleavage, and oxidation (33). In the well-studied anaerobic benzoyl-CoA degradation pathways, crotonase-like enzymes catalyze the addition of water to cyclic mono- or di-enoyl-CoA compounds, as well as the subsequent ring-opening hydrolysis (15, 34–37). Phylogenetic analysis of amino acid sequences showed that ThnA clusters with the *Rhodopseudomonas* type monoenoyl-CoA hydratase (cyclohex-1-ene-1-carboxyl-CoA hydratase) (34), whereas ThnL is more closely related to the *Thauera*/*Geobacter*/*Magnetospirillum* type dienoyl-CoA hydratase (cyclohexa-1,5-dienecarbonyl-CoA hydratase) (16, 36, 38). However, ThnL did not exhibit hydration activity toward the dienoyl-CoA compound hexahydro-2-naphthoyl-CoA in this study. Additionally, ThnM and ThnU belong to the *bbsH* branch of crotonase-like enzymes, which includes benzylidenesuccinyl-CoA hydratase, responsible for adding water to the linear-branched side chain of benzylidenesuccinyl-CoA (12).

A previous study identified the hydration of crotonyl-CoA by a heteromeric ThnHI complex (39). However, phylogenetic analysis showed that ThnH and ThnI did not cluster with crotonyl-CoA hydratases, but instead grouped with MaoC-like dehydratases, suggesting that ThnHI catalyzes a so far unidentified reaction in the naphthalene degradation pathway.

### Active site analysis

Members of the crotonase superfamily catalyze different reactions but share a common catalytic mechanism involving the stabilization of thioester enolate anion intermediates through an oxyanion hole, a conserved pocket formed mainly by backbone amide groups that hydrogen-bond to and stabilize the negatively charged carbonyl oxygen during catalysis (40, 41). The key residues that contribute to the formation of this oxyanion hole are conserved across the crotonase-like hydratases within the *thn* operon, including ThnA (Ala77 and Gly122), ThnL (Ala66 and Gly111), ThnM (Ile79 and Gly125), and ThnU (Ala78 and Gly123), indicating that these enzymes employ the characteristic crotonase-family enolate-stabilizing mechanism, consistent with their sequence-based assignment to this superfamily.

Crotonase-like hydratases typically possess two catalytic residues. In the best-studied rat mitochondrial crotonase, Glu144 serves as the general base that assists the attack of water on the β-carbon by deprotonating the water molecule to generate a hydroxide, while Glu164 acts as the general acid and protonates the α-carbon (42). In ThnL from N47, the Glu164 equivalent is conserved as Glu134; however, the Glu144 equivalent is replaced by Gly114 in ThnL. Glycine lacks a carboxylate side chain and is therefore unable to act as a general base to activate water for nucleophilic attack or engage in the acid-base chemistry required for the canonical crotonase hydratase mechanism.

The absence of the general-base catalytic glutamate residue in crotonase-type hydratases has previously been reported in AtuE, the isohexenyl-glutaconyl-CoA hydratase from *Pseudomonas aeruginosa* (43), and in many other bacterial genomes (41). In these enzymes, the single remaining catalytic glutamate residue has been proposed to fulfill both roles: enhancing the nucleophilicity of the water molecule and protonating the substrate at the α-carbon (42). Alternatively, in the methylglutaconyl-CoA hydratase (LiuC) from *Pseudomonas putida*, the substrate’s free carboxylate group was hypothesized to functionally replace the missing general-base glutamate residue and activate water for nucleophilic attack; this explains why LiuC cannot hydrate substrates that lack such an additional carboxylate group (41). Notably, the physiological substrate of ThnL, 1-(1-hydroxy-2-carboxycyclohexyl)acetyl-CoA, also contains a free carboxylate group, which could analogously substitute for the absent general-base glutamate and participate in water activation. This mechanism possibly explains the observation in this study that ThnL does not show activity toward hexahydro-2-naphthoyl-CoA. This substrate lacks a free carboxylate group, and its thioester carbonyl is not an ionized carboxylate and therefore cannot serve as a general base. Without a free carboxylate to compensate for the missing catalytic glutamate, the reaction can probably not proceed.

Certain members of the crotonase superfamily catalyze Dieckmann reactions, which are intramolecular Claisen condensations in which the enzymes catalyze the formation of a cyclic β-keto ester from a di-ester or acyl-CoA substrate. In the anaerobic catabolism of benzoate by *Rhodopseudomonas palustris*, the hydrolysis of 2-oxocyclohexanecarboxyl-CoA to pimelyl-CoA is catalyzed by the hydrolase BadI. This reverse Dieckmann reaction is expected to proceed via the addition of water, forming a hydrated ketone that promotes the cleavage of the α- and β*-*carbon bonds via protonation of the enolate anion intermediate (44). In the present study, ThnL was found to catalyze the same hydrolysis of 2-oxocyclohexanecarboxyl-CoA, although its specific activity is only 0.06% of the hydration of 2-carboxycyclohexylideneacetyl-CoA catalyzed by ThnL. Another crotonase superfamily member homologous to BadI is MenB, the 1,4-dihydroxy-2-naphthoyl-CoA synthase that catalyzes a Dieckmann condensation (45). Phylogenetic analysis shows that ThnL does not cluster with MenB or BadI (Fig. 7). Given that MenB and BadI form a phylogenetic cluster associated with Dieckmann-type reactions, whereas ThnL lies outside this group and exhibits only weak activity in this chemistry, the hydrolysis of 2-oxocyclohexanecarboxyl-CoA by ThnL is likely a promiscuous side activity rather than its primary physiological function.

Notably, the other crotonase-like hydratases encoded within the *thn* operon—ThnA, ThnM, and ThnU, which showed no activity in converting 2-carboxycyclohexylideneacetyl-CoA—possess two glutamic acid catalytic residues at the active sites. This observation suggests that these enzymes may specifically catalyze hydration reactions of substrates lacking additional carboxylic acid groups, such as hexahydro-2-naphthoyl-CoA and β-oxooctahydro-2-naphthoyl-CoA, which are intermediates in the upper pathway of naphthalene degradation.

### Insights into the second-ring cleavage of naphthalene

The hydration of 2-carboxycyclohexylideneacetyl-CoA involves a water addition on the branched β-carbon, producing the tertiary alcohol 1-(1-hydroxy-2-carboxycyclohexyl)acetyl-CoA. However, hydratases that act on substrates with a branched β-carbon skeleton are poorly understood. Examples of such enzymes include isohexenylglutaconyl-CoA hydratase (AtuE) and methylglutaconyl-CoA hydratase (LiuC) involved in the catabolism of acyclic terpenes and leucine/isovalerate (41, 46). To accommodate the branched acyl moiety of the substrate, AtuE has evolved a considerably expanded catalytic cavity (43). AtuE and LiuC introduce a hydroxyl group at the tertiary carbon atom of their substrates. Following this, the branched intermediates were supposed to undergo carbon-carbon bond cleavage, releasing acetate or acetyl-CoA, which was expected to be catalyzed by AtuA and LiuE, respectively (46). Based on these findings, two potential strategies for opening the cyclohexane ring of 1-(1-hydroxy-2-carboxycyclohexyl)acetyl-CoA during anaerobic degradation of naphthalene were proposed (17) (Fig. 1). A LiuE-like pathway in N47 involving a putative CoA-transfer and a lyase reaction would release acetate and produce 2-oxocyclohexanecarboxyl-CoA. Although ThnL shows extremely weak hydrolase activity toward 2-oxocyclohexanecarboxyl-CoA observed in the *in vitro* experiment, strain N47 cells did not show growth when this compound was supplied as the sole carbon source. We conclude that the LiuE-like pathway is unlikely to be the actual or preferred cleavage route in N47 cells. In contrast, our results favor an AtuA-like aldolase-mediated ring-opening mechanism between carbons 1 and 2 of the cyclohexane ring of 1-(1-hydroxy-2-carboxycyclohexyl)acetyl-CoA as the more plausible of the two previously proposed pathways (17). In this context, the reaction catalyzed by ThnL, that is, the hydration of 2-carboxycyclohexylideneacetyl-CoA, provides the last reaction preparing for the second-ring cleavage reaction during anaerobic naphthalene degradation.

## Data Availability

The data supporting the study findings are available from the corresponding author upon reasonable request.

## SUPPLEMENTAL MATERIAL

Table S1 aem.00467-26-s0001.docx Primers used in this study.

ASM does not own the copyrights to Supplemental Material that may be linked to, or accessed through, an article. The authors have granted ASM a non-exclusive, world-wide license to publish the Supplemental Material files. Please contact the corresponding author directly for reuse.
