# Supplementary material for: Identification and characterization of the hydratase ThnL preparing for the second-ring cleavage in anaerobic naphthalene degradation
Source: Appl Environ Microbiol. 2026 Sep 23;92(10):e00467-26. doi: 10.1128/aem.00467-26 (PMC13689101; doi:10.1128/aem.00467-26)
Supplement: Supplementary file 1 [file aem.00467-26-s0001.docx]

Table S1. List of primers used in this study

| Gene | Tag | Primer | Sequence (5′→3′) | annealing temperatures |
| --- | --- | --- | --- | --- |
| *thnA* | His6 | forward | ATATTCATGAAATATGAAAATATCATTTATGAGGTAAAG | 63℃ |
|  |  | reverse | ATATCTCGAGTTTACCTGTAAAGACGGGTTTTCTTTTTTC |  |
| *thnH* | His6 | forward | ATATTCATGAAGGAAATCTTATATGATGATCTG | 63℃ |
|  |  | reverse | ATATCTCGAGCTTAGGCCATATCGGACTTAG |  |
| *thnI* | His6 | forward | ATATTCATGAGTTGTTACAGAACTTTTGAAG | 64℃ |
|  |  | reverse | ATATCTCGAGGTTATTTTTAGGGAACGCTGCC |  |
| *thnL* | His6 | forward | ATATCCATGGCTTATAAAAATATATTATACGAGATTG | 63℃ |
|  |  | reverse | ATATCTCGAGCCTTCCCTTCCATACGGGC |  |
| *thnM* | Twin-strep | forward | AGCGCGTCTCCAATGGAAGGGAAGGTAGAGAAA | 72℃ |
|  |  | reverse | AGCGCGTCTCCTCCCCATTCCGTTATATTTAGGCTTTCTTT |  |
| *thnU* | His6 | forward | ATATCCATGGAGTCACAACTGGTTCACTATG | 67℃ |
|  |  | reverse | ATATCTCGAGTTTACATTTATATTCAGGTTTTCTTTTTTCG |  |
